# Return to Work and Work Productivity During the First Year After Cancer Treatment

**DOI:** 10.3389/fpsyg.2022.866346

**Published:** 2022-04-12

**Authors:** Serana Chun Yee So, Danielle Wing Lam Ng, Qiuyan Liao, Richard Fielding, Inda Soong, Karen Kar Loen Chan, Conrad Lee, Alice Wan Ying Ng, Wing Kin Sze, Wing Lok Chan, Victor Ho Fun Lee, Wendy Wing Tak Lam

**Affiliations:** ^1^LKS Faculty of Medicine, Jockey Club Institute of Cancer Care, The University of Hong Kong, Hong Kong, Hong Kong SAR, China; ^2^School of Public Health, Centre for Psycho-Oncology Research and Training, The University of Hong Kong, Hong Kong, Hong Kong SAR, China; ^3^Department of Clinical Oncology, Pamela Youde Nethersole Eastern Hospital, Hong Kong, Hong Kong SAR, China; ^4^Department of Obstetrics and Gynaecology, The University of Hong Kong, Hong Kong, Hong Kong SAR, China; ^5^Department of Clinical Oncology, Princess Margaret Hospital, Hong Kong, Hong Kong SAR, China; ^6^Department of Clinical Oncology, Tuen Mun Hospital, Tuen Mun, Hong Kong SAR, China; ^7^Department of Clinical Oncology, The University of Hong Kong, Kowloon, Hong Kong SAR, China

**Keywords:** cancer survivors, return to work, occupation, illness perception, health-related quality of life, work productivity

## Abstract

**Objectives:**

Working-age cancer patients face barriers to resuming work after treatment completion. Those resuming work contend with reduced productivity arising from persisting residual symptoms. Existing studies of return to work (RTW) after cancer diagnosis were done predominantly in Western countries. Given that employment and RTW in cancer survivors likely vary regionally due to healthcare provision and social security differences, we documented rates and correlates of RTW, work productivity, and activity impairment among Chinese cancer survivors in Hong Kong at one-year post-treatment.

**Methods:**

Of 1,106 cancer patients assessed at six-months post-cancer treatment (baseline), 593 previously worked; detailed work status, psychological distress (HADS), physical symptom distress (MSAS-SF), supportive care needs (SCNS-SF34-C), health-related quality of life (SF12), and illness perception (B-IPQ) were assessed. Six months later (follow-up), work productivity and activity impairment were assessed (WPAI; *n* = 402). Descriptive analyses examined RTW rate. Fully adjusted regressions determined RTW, work productivity, and activity impairment predictors.

**Results:**

At baseline, 39% (232/593) were working, 26% (153/593) on sick leave, and 35% (208/593) were unemployed. Compared to patients returning to work, unemployed participants were older, likely manual/service-oriented workers, and had lower family income, chemotherapy, fewer unmet health system and information needs, poorer physical functioning, and negative illness perceptions. Sick leave participants were likely service-oriented workers, who had head and neck cancer, chemotherapy, and poor physical functioning. At FU, baseline depressive symptoms, physical symptom distress, and negative illness perceptions predicted presenteeism and work productivity loss; gynecological cancer, fewer unmet health system and information needs, and greater unmet sexuality needs predicted absenteeism; physical symptom distress, negative illness perception, and poor physical functioning predicted activity impairment.

**Conclusion:**

Cancer survivors who had more physically demanding jobs and poorer physical functioning delayed RTW. Unmanaged physical symptom and psychological distress hindered work productivity.

## Introduction

There is a large and growing population of cancer survivors worldwide, attributable to early detection and advancement in cancer treatments, as well as to an aging population (Kamal et al., 2017). In estimate, 40% of cancer survivors are aged <65, with 35% being between the ages of 40 and 64, an age when career and work-related issues play a crucial role in their lives (Mehnert, 2011; Butow et al., 2020). With a rising life expectancy, retirement-age thresholds in many counties with high Human Development index (e.g., Europe and the United States) have been extended; it is not uncommon to see older-aged adults fully active in the work force (Mehnert et al., 2013). Thus, returning to work (RTW) after cancer diagnosis and treatments has become a major challenge for this population. Examining influences on RTW after cancer diagnosis can help in focusing cancer rehabilitation on social reintegration. In particular, RTW has been seen by cancer survivors as an indication of reintegration into normal life following cancer diagnosis, which is critical for personal development, identity formation, social recovery, and promoting self-esteem and quality of life, and also as having extrinsic value for making contribution to society and economic benefits (Spelten et al., 2002; Butow et al., 2020; Thandrayen et al., 2022).

Studies are increasingly investigating employment and work-related issues among cancer survivors (Mehnert, 2011). A recent systematic review suggests that the mean RTW rate post-diagnosis was 63.5% ranging from 24 to 94% (Mehnert, 2011). There is evidence that side effects of disease- and treatment-related factors, such as fatigue or pain, may hinder patient RTW (Steiner et al., 2008; Munir et al., 2010; Taskila et al., 2011; Muijen et al., 2013; Kamal et al., 2017). Sociodemographic factors (i.e., older age, female gender, and lower educational achievement) and work-related factors, including poor adjustment to work, have also been associated with impaired RTW (Mujahid et al., 2010; Mehnert, 2011; Duijts et al., 2017). However, existing data regarding RTW after cancer diagnosis has been largely derived from Western European, North American, and Australian populations (Islam et al., 2014). Employment and RTW in cancer survivors are likely to vary greatly across regions, due to differences in healthcare provision, employment policies, and social security (Mehnert et al., 2013). For example, in Hong Kong, up to 120 days paid sick leave days can be accumulated when supported by valid medical certificate (e-Legislation HK, 2015). Employers are legally prevented from terminating the employment contract of ill workers during the paid sickness absence period (e-Legislation HK, 2015). In contrast, there is no government policy mandating paid sick leave in the United States (Mehnert et al., 2013). Specifically, there is no statutory retirement age in Hong Kong other than in the public sector, where the mandatory retirement age is 60–65. Given that Hong Kong has very little pension provision other than the meager Mandatory Provident Fund, many retirement-age people rely on extended working for financial support after retirement. Some personnel re-enter the private sector as a consultant, freelance, or part-time workers after their formal retirement. In 2018, the employment rate of elders aged 65 and above in Hong Kong was 11.7%, which was significantly greater than in some western countries (e.g., German, Italy, and France; Chan and Yip 2019). Therefore, the RTW rate and its determinants might also differ in Hong Kong cancer population. Despite a recent spike in research on Asian populations (Park et al., 2008, 2009; Lee et al., 2017; Su et al., 2018), none has been done in Hong Kong Chinese context. Gathering data from a broader range of cultural, ethnic, and national groups is crucial to build a more complete picture for the development of effective interventions to enhance RTW for cancer survivors everywhere (Lee et al., 2017). Furthermore, despite numerous studies having investigated the rate of RTW and its associated factors among cancer survivors, less attention has been focused on the impact of cancer diagnosis on work productivity including absenteeism (i.e., missing time from work), presenteeism (i.e., reduced performance while at work), and activity impairment on those who return to work. There is some evidence that cancer survivors face a reduction in both work hours and in physical or mental work capacity (Mehnert, 2011). Changes in work productivity often precede work cessation (Bradley et al., 2005; de Boer et al., 2008; Lee et al., 2008) and are important to explore and understand how cancer diagnosis impacts productivity loss.

This study examined RTW and its predictors among Hong Kong Chinese cancer survivors recently completing cancer treatment. Several factors such as demographics, physical impairment (e.g., physical symptoms), and psychosocial resources have been proposed to be related to RTW after cancer diagnosis (Mehnert, 2011), but again, evidence in Chinese cancer populations remains limited. Hence, we tested if RTW is related to demographic factors, specifically age, gender, education level, marital status, family income and occupation, physical impairment including physical symptom distress and health-related quality of life, and psychosocial resources. Moreover, because negative illness perception has been widely associated with poor adaptation to cancer diagnosis (Mickevičienė et al., 2013), potentially delaying work resumption among cancer patients (Chen et al., 2021), we also tested if RTW is predicted by variations in illness perception. Finally, we examined work productivity and activity impairment at one-year post-treatment and its associations with demographic factors, physical impairment, and psychosocial resources.

## Materials and Methods

### Participants and Design

This study is a secondary analysis of a larger local study on cancer survivorship (Zhang et al., 2016). Following ethical approval (ref: UW10-203), Chinese cancer survivors were recruited consecutively from eight Hong Kong public hospital oncology clinics between September 2010 and June 2013 for the original study (Zhang et al., 2016). At each hospital, clinical oncologists identified functionally capable, eligible patients from clinic lists. Eligible participants were immediately approached by a trained research assistant while awaiting consultation. Inclusion criteria in the original study were Cantonese- or Mandarin-speaking Chinese cancer survivors aged 18 or above and who had completed primary treatment in the last 6 months (Zhang et al., 2016). Patients with linguistic or intellectual difficulties were excluded (Zhang et al., 2016). Eligible patients were approached by a trained research assistant at oncology outpatient clinics. After explanation of the study, written consent was obtained from those who agreed to participate. As a baseline, a standardized face-to-face, questionnaire-based interview was then carried out at the oncology outpatient clinic. A follow-up interview was conducted at six-months post-baseline. All interviews were conducted in Cantonese or Mandarin by a trained research assistant.

For the purpose of the current study, we only included participants who had paid- or self-employment at the time of diagnosis. Since there is no statutory retirement age for employees in the private sector in Hong Kong, all adult patients were included in this study.

### Measures

#### Outcome Variables

Apart from RTW assessed at baseline (six-months post-treatment) and six-months post-baseline (follow-up; one-year post-treatment), other outcome variables (i.e., work productivity and activity impairment) were assessed only once at follow-up.

##### Return to Work

RTW was measured as time to RTW after an absence from work due to the diagnosis of cancer including both paid and unpaid time off from work (i.e., time to RTW after sick leave; de Boer et al., 2008; Mehnert, 2013). Patients were asked if they were still on sick leave since the first day of sick leave and indicated the date of RTW. For those who have returned to work, patients were asked if they have changed jobs, job nature, or working hours. For those who did not take time off from work, the time to RTW was set as zero.

##### Work Productivity and Activity Impairment

The Work Productivity and Activity Impairment (WPAI) questionnaire was used to assess loss of work productivity and activity (Reilly et al., 1993). The WPAI consists of six questions measuring four domains including absenteeism (number of work hours missed due to current health condition), presenteeism (the extent the current health condition affects productivity at work), work productivity loss (the extent of current health condition-induced work inability), and activity impairment (the extent that the current health condition affects regular activities other than job-related work). Each of the WPAI outcomes is expressed as a percentage, with higher values indicating greater impairment and productivity loss. The WPAI has been used to assess work productivity loss resulting from chronic health conditions (e.g., irritable bowel syndrome; Reilly et al., 2004; Bushnell et al., 2006). Its discriminative validity and reproducibility have been established, with intraclass correlation coefficients ranging from 0.68 to 0.98 (Reilly et al., 2004; Bushnell et al., 2006).

#### Predictors/Covariates

All potential predictors including physical symptom distress, health-related quality of life, psychosocial resources, and illness perception were assessed once at baseline.

##### Physical Symptom Distress

Physical symptom distress was assessed using the 12-item physical symptom distress subscale from the Chinese version of the Memorial Symptom Assessment Scale Short Form (MSAS-SF; Lam et al., 2008). Participants were asked to indicate any listed symptoms experienced in the past seven days and rated associated distress on a 5-point Likert response options: “Not at all,” “A little bit,” “Somewhat,” “Quite a bit,” and “Very much.” Mean item scores range from 0 to 4, with higher scores reflecting greater physical symptom distress (Lam et al., 2008). The Chinese version of MSAS-SF has demonstrated good validity and reliability in Chinese cancer populations, with Cronbach’s alpha ranged from 0.84 to 0.91 (Lam et al., 2008).

##### Health-Related Quality of Life

The Medical Outcomes Study 12-item Short-Form Health Survey (SF12) is a generic measure of health-related quality of life and evaluates physical functioning, role physical, bodily pain, general health, vitality, social functioning, role emotional, and mental health dimensions (John et al., 1996; Lam et al., 2005). The 12 items are used to derive a physical component score (PCS) and a mental component score (MCS; John et al., 1996; Lam et al., 2005). The scores were transformed to a 0–100 scale, higher scores indicating better physical and mental functioning. The Hong Kong Chinese version has been developed and validated for use in Hong Kong Chinese population including those with chronic diseases such as heart disease and stroke; the SF-12 PCS and MCS explained 82 and 89% of the total variance in the SF-35, the long-form of the SF-12, respectively (Lam et al., 2005). The Chinese version of SF-12 overall demonstrated an acceptable internal consistency among Chinese patients with advanced cancer and spousal caregivers, with a Cronbach’s alpha of 0.81 (Li et al., 2016).

##### Psychosocial Resources

We conceptualized psychological distress and perceived supportive care needs as reflecting the inverse, an absence or inadequacy, of psychosocial resources.

Psychological distress was assessed using the 14-item Hospital Anxiety and Depression Scales (HADS; Snaith and Zigmond, 1986). Comprised of 27 item subscales that measure symptoms of anxiety and depression, each item is rated on a four-point scale. Total scores for each subscale range from 0 to 21, with higher scores indicating greater distress. The Chinese version of the HADS has been validated in local settings with general hospital in-patients, with satisfactory internal consistency for both anxiety (Cronbach’s α = 0.86) and depression (Cronbach’s α = 0.82; Leung et al., 1999).

The Chinese version of the Supportive Care Needs Survey Short Form (SCNS-SF34-C) was adopted to assess type and magnitude of unmet need (Au et al., 2011; Li et al., 2013). The original SCNS-SF-34-C has robust psychometric properties and is widely used among cancer patients internationally (Choi et al., 2020). Patients’ perceived need for help is measured across five domains: health system and information (11 items); psychological (10 items); physical and daily living (five items); sexuality (three items); and patient care and support needs (five items). Patients rate the intensity of each need over the past month for each item using five-point Likert scales (Spelten et al., 2002; Mehnert, 2011; Mehnert et al., 2013; Kamal et al., 2017; Butow et al., 2020): 1 = No need: not applicable; 2 = No need: satisfied; 3 = Low need; 4 = Moderate need; and 5 = High need (“No need: not applicable” reflects that patients perceived “this was not a problem for me as a result of having cancer”; “No need: satisfied” indicates that “the patient did need help with that item but their need for help was satisfied at the time”; “Low need” reflects that “This item causes the patients some concern or discomfort (But) They have little need for additional help.”; “Moderate need” reflects “This item causes the patient concern or discomfort. They have some need for additional help.”; and “High need” reflects that “This item causes patients concern or discomfort. They have a strong need for additional help…”) Scores were converted to standardized Likert summated scores which range from 0 to 100 when calculating domain scores, with higher scores indicating greater perceived unmet need (Choi et al., 2020). The SCNS-SF34-C has demonstrated acceptable content validity and internal reliability in Chinese cancer patients (Cronbach’s α = 0.82–0.92; Au et al., 2011; Li et al., 2013).

##### Illness Perception

The Chinese version of the nine-item Brief Illness Perception Questionnaire (B-IPQ) was used to assess cognitive and emotional representations of illness (Zhang et al., 2016). Five items, Consequences, Timeline, Personal control, Treatment control, and Identity reflect cognitive aspects of illness representations, whereas two items, Concern and Emotions, reflect emotional aspects of illness representations (Zhang et al., 2016). Illness comprehensibility was assessed by one item (Coherence). All items are rated using 0 to 10 Likert scales except one open-ended casual question, which asks the patients to suggest three most important factors that they believe have caused their illness. Higher IPQ scores indicate more threatening perceptions of the illness (Zhang et al., 2016). The Chinese version of B-IPQ has been validated in Chinese cancer population, with acceptable internal consistency (Cronbach’s α = 0.56–0.78; Fan et al., 2013).

##### Demographic and Clinical Information

At baseline, patients’ sociodemographic data (age, education, marital status, family income, and job type) were collected through a face-to-face, questionnaire-driven interview at baseline, while clinical data (cancer type, stage, time since diagnosis, time since surgery, and adjuvant therapy) were extracted from patients’ medical record using a standard protocol.

### Data Analyses

All data analyses were conducted using the Statistical Package for Social Sciences version 26.0 (SPSS, Chicago, IL, United States). Descriptive statistics were used to describe the characteristics of the study sample, as well as the RTW status (returned to work, on sick leave, and unemployed). Work resumption was the primary endpoint of all analyses. In order to test covariates with RTW status, univariate analyses, using one-way ANOVA, were conducted to assess the relationship of study correlates (supportive care needs, physical symptom distress, psychological distress, health-related quality of life, and illness perception) with RTW status. We also used univariate analyses to identify significant demographic and clinical correlates of RTW status. Next, multinomial regression analysis was performed to test the extent to which RTW status can be differentiated by the proposed correlates after adjustment for significant demographic and clinical factors. Odds ratios are reported together with 95% confidence intervals. A similar analytical approach was adopted to examine the role of baseline factors in predicting work productivity and activity impairment at six-months follow-up. Multiple linear regression analysis was used to examine which, if any, of the proposed factors predicted absenteeism, presenteeism, work productivity loss, and activity impairment. Complete-case analysis was used to handle missing data.

## Results

**Figure 1:**
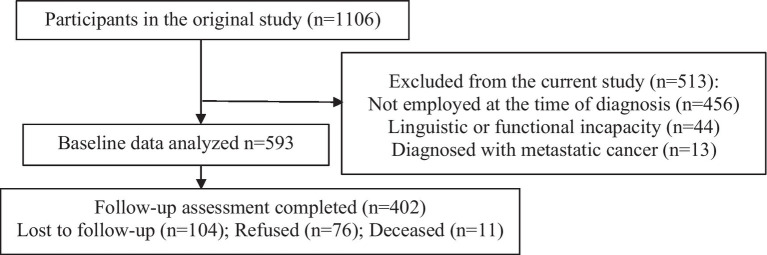
Sampling structure and attrition pattern of the study.

A total of 1,106 cancer survivors gave informed consent to participate in a larger study of cancer survivorship (Zhang et al., 2016). Of these, 513/1106 participants were ineligible for this sub-study due to not holding active employment at the time of diagnosis (*n* = 456), linguistic or functional incapacity (*n* = 44), or diagnosis of metastatic cancer (*n* = 13; Figure 1). Hence, the baseline analyses were performed on the remaining sample of 593. Of these, 402/593 participants (68%) completed the one-year post-treatment questionnaire. Demographic factors and clinical characteristics did not differ significantly between those who completed and those who did not complete the six-month follow-up assessment, except in one category: cancer type (patients who suffered from “Others” cancer types, for example. Lung, prostate, or leukemia for which insufficient numbers allowed independent categorization).

### Return to Work Status

Table 1 summarizes the demographic and clinical characteristics of the study sample, and their RTW status. At baseline (six-months post-treatment), 39% (232/593) of participants had returned to work; 26% (153/593) were on paid or unpaid sick leave; and 35% (208/593) were unemployed. At follow-up (one-year post-treatment), 63% (255/402) of participants had returned to work; 33% (134/402) were unemployed; and 3% remained on paid or unpaid sick leave (13/402). Of those on sick leave at baseline, most (71%) had resumed work at one-year post-treatment, but 23% were unemployed. At Baseline, participants who had resumed work (mean 49 years, SD = 10.17) were significantly younger than those who were unemployed (mean 53 years, SD = 9.44; *t* = −4.118, *p* < 0.001); no significant difference was found in age between those who had resumed work and those on sick leave (*p* = 0.43). A significantly greater proportion of patients with tertiary education [62% (61/98)] had resumed work as compared to those with secondary [39% (129/330)] or primary education [25% (42/165); *χ*^2^ = 17.93, *p* < 0.001]. Of patients with primary education, 57% (94/165) were unemployed.

**Table 1:** Summary of demographic and clinical characteristics (RTW status at baseline, *N* = 593).

	All subjects *N* = 593	Returned to work *N* = 232 (39%)	Sick Leave *N* = 153 (26%)	Unemployed *N* = 208 (35%)
Demographic factors Mean Age ± SD^++^	50.24 ± 9.50	49.08 ± 10.17	48.31 ± 7.57	52.94 ± 9.44
Gender^**^				
Male Female	215 (36.3) 378 (63.7)	75 (32.3) 157 (67.7)	62 (40.5) 91 (59.5)	78 (37.5) 130 (62.5)
Education level^**^^,^ ^++^				
No formal education or primary education Secondary education Tertiary education	165 (27.8) 330 (55.7) 98 (16.5)	42 (18.1) 129 (55.6) 61 (26.3)	29 (18.9) 102 (66.7) 22 (14.4)	94 (45.2) 99 (47.6) 15 (7.2)
Marital status^**^				
Single Married Divorced or Widowed Missing	89 (15.0) 434 (73.2) 69 (11.6) 1 (0.2)	43 (18.5) 162 (69.8) 27 (11.7) –	29 (19.0) 111 (72.5) 13 (8.5) –	17 (8.2) 161 (77.4) 29 (13.9) 1 (0.5)
Job type^**^^,^ ^++^				
White collar (professional/business) Blue collar Services Missing	250 (42.2) 101 (17.0) 240 (40.5) 2 (0.3)	137 (59.0) 23 (9.9) 72 (31.1) –	69 (45.1) 25 (16.3) 57 (37.3) 2 (1.3)	44 (21.1) 53 (25.5) 111 (53.4) –
Total monthly family income (HK$7.8 = US$1.00)^++^				
<HK$10000 HK$10001–$20,000 HK$20001–$40,000 >HK$40000 Missing	140 (23.5) 184 (31.0) 168 (28.4) 81 (13.7) 20 (3.4)	27 (11.7) 70 (30.2) 85 (36.6) 46 (19.8) 4 (1.7)	27 (17.7) 50 (32.7) 52 (33.9) 23 (15.0) 1 (0.7)	86 (41.3) 64 (30.8) 31 (14.9) 12 (5.8) 15 (7.2)
Clinical factors (%)				
Cancer type^++^ Breast Head and Neck (including nasopharyngeal) Colorectal Gynecological Others^^^	239 (40.3) 139 (23.5) 77 (13.0) 66 (11.1) 72 (12.1)	113 (48.7) 34 (14.7) 31 (13.4) 24 (10.3) 30 (12.9)	58 (37.9) 53 (34.6) 14 (9.2) 19 (12.4) 9 (5.9)	68 (32.7) 52 (25.0) 32 (15.4) 23 (11.0) 33 (15.9)
Stage of cancer				
Stage 0 or I Stage II Stage III Missing	137 (23.1) 218 (36.8) 192 (32.3) 46 (7.8)	63 (27.2) 86 (37.1) 66 (28.4) 17 (7.3)	32 (20.9) 59 (38.6) 51 (33.3) 11 (7.2)	42 (20.2) 73 (35.1) 75 (36.1) 18 (8.6)
Previous treatment				
Surgery^**^^,^ ^++^ Chemotherapy Targeted therapy^*^^,^ ^+^ Radiation therapy^*^^,^ ^+^	431 (72.7) 446 (75.2) 40 (6.7) 471 (79.4)	192 (82.8) 163 (70.3) 21 (9.1) 188 (81.0)	95 (62.1) 119 (77.8) 5 (3.3) 118 (77.1)	144 (69.2) 164 (78.8) 14 (6.7) 165 (79.3)
Current treatment Hormonal therapy^*^^,^ ^+^	151 (25.5)	73 (31.5)	32 (20.9)	46 (22.1)
Time since initial diagnosis (months)	9.46 ± 6.86	10.55 ± 7.94	7.13 ± 3.74	9.94 ± 6.94
	*N* = 402	*N* = 162	*N* = 109	*N* = 131
Return to work status a one-year post-treatment				
Returned to work	255 (63.4)	151 (93.2)	77 (70.6)	27 (20.6)
Sick leave	13 (3.3)	2 (1.2)	7 (6.4)	4 (3.1)
Unemployed	134 (33.3)	9 (5.6)	25 (23.0)	100 (76.3)

Others^^^ include lung (n = 24), prostatic (n = 11), leukaemia (n = 10), liver (n = 8), and others (n = 19).

* *p* < 0.05 was found between returned to work group and sick leave group.

** *p* < 0.01 was found between returned to work group and sick leave group.

+ *p* < 0.05 was found between returned to work group and unemployed group.

++ *p* < 0.01 was found between returned to work group and unemployed group.

### Return to Work Correlates at Baseline

Table 2 summarizes the descriptive data for the measures of supportive care needs (SCNS-SF34-C), physical symptom distress (MSAS-SF), psychological distress (HADS), illness perception (B-IPQ), and health-related quality of life (SF12) for all subjects, and by RTW status. Using participants who had returned to work as the reference group, ANOVA with post-hoc Bonferroni multiple comparisons showed that the SCNS-SF34-C Health System and Information and Physical and Daily Living domains, HADS Depression subscale, MSAS-SF Physical symptom distress, B-IPQ Total, and SF12 Physical and Mental component scores differed significantly by RTW status at baseline. Those who had resumed work reported significantly lower physical and daily living needs (*p* < 0.001), fewer depressive symptoms (*p* < 0.001), lower physical symptom distress (*p* < 0.001), better physical (*p* < 0.001) and psychological functioning (*p* < 0.001), and more positive illness perceptions (*p* < 0.001) than those who were on sick leave. On the other hand, those who were unemployed had significantly fewer health system and information needs (*p* = 0.041), more physical and daily living needs (*p* = 0.032) more depressive symptoms (*p* < 0.001), poorer physical (*p* = 0.017) and psychological (*p* < 0.001) functioning, and more negative illness perceptions (*p* < 0.001) than those who had resumed work.

**Table 2:** Baseline descriptive data of subjects with different RTW status (*N* = 593).

Variables Mean ± SD	All subjects *N* = 593	Returned to work *N* = 232 (39%)	Sick Leave *N* = 153 (26%)	Unemployed *N* = 208 (35%)
SCNS-SF34-C				
Health system and information^+^ Psychological Physical and daily living^**^^,^ ^+^ Sexuality Patient care and support	36.05 ± 20.96 13.89 ± 14.70 15.27 ± 13.48 6.32 ± 11.95 24.97 ± 20.56	37.86 ± 21.01 12.03 ± 13.42 12.66 ± 11.80 5.60 ± 11.36 25.93 ± 20.58	37.58 ± 21.15 15.53 ± 15.72 18.33 ± 15.02 5.61 ± 9.74 23.95 ± 19.77	32.92 ± 20.50 13.89 ± 14.70 15.92 ± 13.53 7.65 ± 13.86 24.66 ± 21.16
HADS				
Anxiety Depression^**^^,^ ^++^	2.62 ± 3.03 3.25 ± 3.29	2.44 ± 2.98 2.41 ± 2.70	2.82 ± 2.94 3.74 ± 3.46	2.66 ± 3.16 3.85 ± 3.58
MSAS-SF				
Physical symptom distress^**^	0.51 ± 0.53	0.40 ± 0.41	0.68 ± 0.64	0.50 ± 0.51
Total B-IPQ^**^^,^ ^++^	34.36 ± 12.56	31.00 ± 12.58	36.17 ± 11.72	36.84 ± 12.36
SF12				
Physical component score^**^^,^ ^+^ Mental component score^**^^,^ ^++^	46.56 ± 8.36 44.21 ± 10.66	48.32 ± 7.73 46.81 ± 9.96	44.52 ± 8.81 42.60 ± 10.37	46.11 ± 8.34 42.49 ± 11.09

** *p* < 0.01 was found between returned to work group and sick leave group.

+ *p* < 0.05 was found between returned to work group and unemployed group.

++ *p* < 0.01 was found between returned to work group and unemployed group.

Next, multinominal regression was performed to compare physical symptom and psychological distress, supportive care needs, health-related quality of life as well as illness perceptions with RTW status, adjusted for age, job type, family income, previous chemotherapy, and cancer type only due to multicollinearity (Table 3). The model was significant (*χ*^2^ = 240.64, *p* < 0.001), accounting for 35% of variation in RTW status (Cox and Snell *R*^2^). Compared to those who had resumed work, those on sick leave were more likely to be service-oriented workers (OR = 1.75, 95% Cl = 1.02–2.99, *p* = 0.04), diagnosed with head and neck or nasopharyngeal cancer (OR = 2.40, 95% Cl = 1.22–4.72, *p* = 0.01), who had undergone chemotherapy (OR = 0.51, 95% Cl = 0.29–0.91, *p* = 0.02) and reported poorer physical functioning (OR = 0.96, 95% Cl = 0.93–1.00, *p* = 0.03). Those who were unemployed were more likely to be older (OR = 1.03, 95% Cl = 1.01–1.06, *p* = 0.02), manual-labor (OR = 3.16, 95% Cl = 1.54–6.51, *p* = 0.002), or service-oriented workers (OR = 3.00, 95% Cl = 1.73–5.22, *p* < 0.001), having lower family incomes (OR = 0.23, 95% Cl = 0.13–0.41, *p* < 0.001; OR = 0.15, 95% Cl = 0.07–0.31, *p* < 0.001), had undergone chemotherapy (OR = 0.43, 95% Cl = 0.24–0.77, *p* = 0.004), reported slightly fewer health system and information needs (OR = 0.98, 95% Cl = 0.97–1.00, *p* = 0.04), but poorer physical functioning (OR = 0.96, 95% Cl = 0.93–1.00, *p* = 0.04) and more negative illness perceptions (OR = 1.04, 95% Cl = 1.02–1.07, *p* < 0.001).

**Table 3:** Baseline multinominal regression of RTW status (six-months post-treatment; *N* = 593; returned to work, *N* = 232 as reference).

Variables	Sick Leave *N* = 153			Unemployed *N* = 208		
	OR	95% CI	*p*-value	OR	95% CI	*p*-value
Age	0.99	0.97–1.02	0.41	1.03	1.01–1.06	0.02^*^
Job type						
White collar (professional/business) Blue collar Service	Referent 2.08 1.75	Referent 0.99–4.36 1.02–2.99	Referent 0.05 0.04^*^	Referent 3.16 3.00	Referent 1.54–6.51 1.73–5.22	Referent 0.002^**^ <0.001^**^
Monthly family income (HK$7.8 = US$1.00)						
Less than HK$10000 HK$10001-HK$30000 HK$30001-above HK$40000	Referent 0.83 0.99	Referent 0.41–1.66 0.46–2.15	Referent 0.59 0.96	Referent 0.23 0.15	Referent 0.13–0.41 0.07–0.31	Referent <0.001^**^ <0.001^**^
Received chemotherapy Did not receive chemotherapy	Referent 0.51	Referent 0.29–0.91	Referent 0.02^*^	Referent 0.43	Referent 0.24–0.77	Referent 0.004^**^
Cancer type						
Breast Head and Neck (including nasopharyngeal) Colorectal Gynecological Others^^^	Referent 2.40 0.78 2.12 0.69	Referent 1.22–4.72 0.35–1.75 0.98–4.60 0.27–1.76	Referent 0.01^*^ 0.54 0.06 0.44	Referent 2.02 0.82 1.96 1.68	Referent 0.98–4.15 0.38–1.74 0.87–4.40 0.77–3.64	Referent 0.06 0.60 0.11 0.19
SCNS-SF34-C						
Health system and information Psychological Physical and daily living Sexuality Patient care and support	1.00 1.00 1.01 0.99 0.99	0.98–1.01 0.98–1.03 0.99–1.04 0.96–1.01 0.97–1.00	0.70 0.90 0.26 0.27 0.08	0.98 1.00 1.01 1.02 1.00	0.97–1.00 0.97–1.02 0.98–1.03 1.00–1.05 0.99–1.02	0.04^*^ 0.91 0.63 0.06 1.00
HADS						
Anxiety Depression	0.93 1.04	0.83–1.05 0.94–1.16	0.23 0.45	0.91 1.11	0.81–1.03 1.00–1.24	0.14 0.06
MSAS-SF						
Physical symptom distress	1.24	0.65–2.38	0.51	0.50	0.25–1.00	0.05
Total B-IPQ	1.02	1.00–1.05	0.11	1.04	1.02–1.07	0.001^**^
SF12						
Physical component score Mental component score	0.96 0.98	0.93–1.00 0.95–1.01	0.03^*^ 0.20	0.96 0.98	0.93–1.00 0.95–1.01	0.04^*^ 0.16
Model statistics						
*X*^2^ *p*-value	240.64 <0.001^**^					

Others^^^ include lung (n = 24), prostatic (n = 11), leukaemia (n = 10), liver (n = 8), and others (n = 19).

* *p* < 0.05;

** *p* < 0.01.

### Follow-Up Return to Work Status Correlates (One-Year Post-treatment)

Table 4 shows the multinominal regression comparing physical symptom distress, psychological distress, supportive care needs, health-related quality of life and illness perceptions, adjusted for age, job type, family income, chemotherapy history, and cancer type, by RTW status at follow-up (one-year post-treatment).

**Table 4:** Multinominal regression of RTW status at follow-up (one-year post-treatment; *N* = 402; returned to work, *N* = 255 as reference).

Variables	Sick Leave *N* = 13			Unemployed *N* = 134		
	OR	95% CI	*p*-value	OR	95% CI	*p*-value
Age	1.001	0.925–1.08	0.98	1.04	1.01–1.08	0.007^**^
Job type						
White collar (professional/business) Blue collar Service	Referent 1.089 0.591	Referent 0.167–7.09 0.117–2.99	Referent 0.93 0.53	Referent 1.89 1.32	Referent 0.89–3.99 0.74–2.33	Referent 0.10 0.35
Monthly family income (HK$7.8 = US$1.00)						
Less than HK$10000 HK$10001-HK$30000 HK$30001-above HK$40000	Referent 0.458 0.259	Referent 0.09–2.29 0.03–2.25	Referent 0.34 0.22	Referent 0.32 0.16	Referent 0.18–0.58 0.08–0.35	Referent <0.001^**^ <0.001^**^
Received chemotherapy Did not receive chemotherapy	Referent 3.18	Referent 0.52–19.43	Referent 0.21	Referent 1.44	Referent 0.78–2.66	Referent 0.24
Cancer type						
Breast Head and Neck (including nasopharyngeal) Colorectal Gynecological Others^^^	Referent 9.46 1.62 4.66 –	Referent 1.28–69.73 0.11–23.96 0.51–42.99 –	Referent 0.03^*^ 0.73 0.18 –	Referent 1.12 1.10 1.40 1.40	Referent 0.53–2.36 0.50–2.41 0.63–3.13 0.56–3.46	Referent 0.77 0.82 0.41 0.47
SCNS-SF34-C						
Health system and information Psychological Physical and daily living Sexuality Patient care and support	1.01 1.00 1.06 1.03 0.97	0.97–1.05 0.97–1.05 0.99–1.14 0.97–1.11 0.93–1.02	0.64 0.92 0.09 0.35 0.23	1.00 1.01 1.00 1.02 1.00	0.98–1.02 0.99–1.04 0.97–1.03 1.00–1.05 0.99–1.02	0.99 0.33 0.93 0.05 0.74
HADS						
Anxiety Depression	0.93 1.31	0.64–1.35 0.99–1.73	0.71 0.06	0.92 1.02	0.81–1.05 0.91–1.14	0.21 0.80
MSAS-SF						
Physical symptom distress	0.67	0.12–3.82	0.65	0.66	0.32–1.35	0.25
Total B-IPQ	0.98	0.91–1.05	0.48	1.03	1.00–1.05	0.05
SF12						
Physical component score Mental component score	1.05 1.06	0.94–1.17 0.97–1.16	0.38 0.19	0.97 1.00	0.93–1.01 0.977–1.03	0.10 0.91
Model statistics						
*X*^2^ *p-*value	100.85 <0.001^**^					

* *p* < 0.05;

** *p* < 0.01.

Others^^^ include lung (n = 24), prostatic (n = 11), leukaemia (n = 10), liver (n = 8), and others (n = 19).

The model accounted for 23% of variation in RTW status (Cox and Snell *R*^2^; *χ*^2^ = 100.85, *p* < 0.001). At follow-up, that is one-year post-treatment, those who were on sick leave and unemployed did not report significant differences relative to those who had resumed work in terms of physical symptoms, psychological distress, supportive care needs, health-related quality of life, and illness perceptions after adjustment for demographic and clinical characteristics. However, those who were still on sick leave were more likely to have a diagnosis of head and neck or nasopharyngeal cancer (OR = 9.46, 95% Cl = 1.28–69.73, *p* = 0.03), whereas those who were unemployed were more likely to be older (OR = 1.04, 95% Cl = 1.01–1.08, *p* = 0.007) and have lower family incomes (OR = 0.32, 95% Cl = 0.18–0.58, *p* < 0.001; OR = 0.16, 95% Cl = 0.08–0.35, *p* < 0.001), when compared with those resuming work.

### Work Productivity and Activity Impairment at Follow-Up

Table 5 illustrates the descriptive data of work productivity (i.e., absenteeism, presenteeism, and work productivity loss) and activity impairment at follow-up (one-year post-treatment). Only those who had resumed work (*n* = 255) were able to report their working hours, work hours missed, and perceived work productivity loss. Hence, absenteeism, presenteeism, and work productivity loss are not applicable for those who were on sick leave or unemployed. Overall, while absenteeism was minimal (3.7%), the impacts on presenteeism (17.2%) and work productivity loss (19.5%) were more apparent. In terms of activity impairment, those who were unemployed (mean 28.7%, SD = 25.50) reported significantly greater activity impairment (*p* < 0.001) compared to those resuming work (mean 17.4%, SD = 21.80).

**Table 5:** Descriptive data of work productivity and activity impairment with different RTW status at follow-up (one-year post-treatment; *N* = 402).

Variables Mean ± SD	All subjects *N* = 402	Returned to work *N* = 255	Sick Leave *N* = 13	Unemployed *N* = 134
WPAI				
Absenteeism	4.09 ± 13.32	3.69 ± 11.77	–	–
Presenteeism	17.11 ± 21.62	17.19 ± 21.64	–	–
Work productivity	19.80 ± 23.94	19.54 ± 23.42	–	–
Activity impairment^**^	21.37 ± 23.85	17.42 ± 21.80	20.83 ± 27.78	28.73 ± 25.50

** *p* < 0.01 was found between returned to work group and unemployed group.

Multiple regression analysis was used to assess baseline predictors of later (follow-up) absenteeism, presenteeism, work productivity loss, and activity impairment at one-year post-treatment (Table 6). Patients diagnosed with gynecological cancers (*B* = 0.16, 95% CI = 0.93–13.41, *p* = 0.025) reporting at baseline more sexuality unmet needs (*B* = 0.19, 95% CI = 0.59–3.78, *p* = 0.008), and with fewer baseline health system and information unmet needs (*B* = −0.23, 95% CI = −0.61 to −0.08, *p* = 0.011) were likely to report greater absenteeism. Both presenteeism and work productivity loss were significantly predicted by greater depressive symptoms (*B* = 0.25, 95% CI = 0.53–3.05, *p* = 0.006; *B* = 0.23, 95% CI = 0.40–3.22, *p* = 0.012, respectively), physical symptom distress (*B* = 0.28, 95% CI = 4.36–20.69, *p* = 0.003; *B* = 0.30, 95% CI = 5.88–24.16, *p* = 0.001, respectively), and negative illness perceptions (*B* = 0.23, 95% CI = 4.36–20.69, *p* = 0.003; *B* = 0.20, 95% CI = 0.10–0.70, *p* = 0.01, respectively) at baseline. Activity impairment was more likely to be reported by service-oriented workers (*B* = 0.12, 95% CI = 1.26–9.55, *p* = 0.011) and was significantly predicted by greater physical symptom distress (*B* = 0.13, 95% CI = 0.14–12.84, *p* = 0.045), negative illness perceptions (*B* = 0.16, 95% CI = 0.08–0.55, *p* = 0.009), and poor physical functioning (*B* = −0.146, *p* = 0.017, 95% CI = −0.76 to 0.07) at baseline.

**Table 6:** Multiple regression of work productivity and activity impairment (WPAI) at one-year post-treatment (*N* = 255).

Variables	Absenteeism (*N* = 255)		Presenteeism (*N* = 255)		Work productivity loss (*N* = 255)		Activity impairment (*N* = 402)	
	B	SE	B	SE	B	SE	B	SE
Age	0.051	0.104	−0.067	0.153	−0.039	0.172	−0.041	0.130
Job type								
White collar (professional/business) Blue collar Service	Referent 0.018 −0.049	Referent 3.574 2.034	Referent −0.019 0.053	Referent 5.280 3.006	Referent −0.016 0.026	Referent 5.908 3.363	Referent 0.085 0.122^*^	Referent 3.663 2.628
Family income (HK$7.8 = US$1.00)								
Less than HK$10000 HK$10001-HK$30000 HK$30001-above HK$40000	Referent 0.027 0.012	Referent 1.971 2.844	Referent 0.031 0.110	Referent 2.914 4.206	Referent 0.028 0.098	Referent 3.259 4.701	Referent −0.038 0.061	Referent 2.806 2.874
Received chemotherapy Did not receive chemotherapy	Referent 0.027	Referent 2.149	Referent −0.044	Referent 3.176	Referent −0.040	Referent 3.552	Referent −0.050	Referent 2.747
Cancer type								
Breast Head and Neck (including nasopharyngeal) Colorectal Gynecological Others^	Referent −0.006 −0.047 0.162^*^ −0.071	Referent 2.684 3.052 3.167 3.515	Referent 0.012 0.006 0.014 0.061	Referent 3.962 4.506 4.761 5.189	Referent −0.006 −0.019 0.079 0.024	Referent 4.437 5.046 5.236 5.811	Referent −0.060 −0.003 −0.019 0.046	Referent 3.468 3.639 3.735 4.177
SCNS-SF34-C								
Health system and information Psychological Physical and daily living Sexuality Patient care and support	−0.234^*^ 0.001 −0.092 0.192^**^ 0.066	0.134 0.274 0.544 0.809 0.327	−0.042 −0.111 0.048 0.047 0.134	0.199 0.405 0.805 1.194 0.484	−0.081 −0.067 −0.001 0.091 0.105	0.222 0.453 0.900 1.337 0.541	0.047 −0.010 0.006 0.066 −0.091	0.171 0.306 0.599 0.802 0.381
HADS								
Anxiety Depression	−0.004 0.068	0.470 0.433	−0.170 0.249^**^	0.695 0.639	−0.131 0.226^*^	0.776 0.716	−0.023 0.117	0.601 0.521
MSAS-SF								
Physical symptom distress	0.178	2.805	0.275^**^	4.143	0.297^**^	4.637	0.134^*^	3.299
Total B-IPQ	−0.006	0.093	0.231^**^	0.137	0.204^*^	0.153	0.160^**^	0.119
SF12								
Physical component score Mental component score	−0.084 −0.017	0.141 0.121	−0.111 0.066	0.208 0.179	−0.103 0.065	0.233 0.199	−0.146^*^ −0.020	0.174 0.147
Model statistics								
*R*^2^ *p*-value	0.164 0.011		0.298 <0.001		0.283 <0.001		0.212 <0.001	

Others^^^ include lung (n = 24), prostatic (n = 11), leukaemia (n = 10), liver (n = 8), and others (n = 19). B, standard coefficient beta; SE, standardized error.

* *p* < 0.05;

** *p* < 0.01.

## Discussion

In the current study, only 2-in-5 workers had resumed work at six-months post-treatment (baseline), while at one-year post-treatment (follow-up), 3-in-5 had returned to active employment, given the mean time since initial diagnosis of 9.46 ± 6.86 (months). The observed RTW rate at one-year post-treatment compares closely to rates reported in a previous systematic review of both Caucasian and non-Caucasian cancer patients, of 64% at the first year of diagnosis (Mehnert, 2011). RTW rates among cancer populations have been reported in other Asian populations. A recent systematic review of 12 RTW studies in the Japanese cancer population reported RTW rates of between 54 and 95% with different cancer types (Ota et al., 2019). However, the assessment points for RTW rates in each study were not given, making comparison difficult. Two studies from Korea reported considerably lower RTW rates than observed in the current study (Ahn et al., 2009; Lee et al., 2017); one study reported a 26% RTW rate among participants, half of whom had been diagnosed for more than 5 years (Lee et al., 2017); and the other study reported a 37% RTW rate in patients at 3-years post-cancer diagnosis (Ahn et al., 2009). Again, the RTW rates in these studies are not directly comparable to the current study, as the investigators used different assessment points. Also, given that these two studies only involved breast cancer survivors (Ahn et al., 2009; Lee et al., 2017), the findings may not be generalizable to other cancer types. The current study provides insightful information by capturing RTW rate among various cancer types at early stage of RTW situation, i.e., six-months post-treatment. While most previous studies only examined RTW status at one-year post-diagnosis, the current study enables comparison between factors associated with RTW status right after cancer treatments (six-months post-treatment) and after the initial recovery stage following primary cancer treatments (one-year post-treatment). More importantly, assessing RTW rates at six-months and one-year post-treatment revealed that those who returned to work at six-months post-treatment were more likely to stay in work, while those on sick leave during the six-months post-treatment period were less likely to return to work at one-year post-treatment. This highlights an important clinical implication, namely, that future RTW studies need to consider the determinants of early RTW, and the design and implementation of interventions to facilitate early RTW.

Consistent with previous studies (Cooper et al., 2013; Muijen et al., 2013), older age was significantly associated with unemployment at six-months and one-year post-treatment. Older patients seem more likely to retire from their previous employment after a cancer diagnosis. For those approaching retirement age at the time of cancer diagnosis, physical restrictions following the illness may have accelerated their retirement decision (Lee et al., 2008), or the illness may simply have been taken as a prompt to retire. After all, even though a cancer diagnosis may not directly lead to unemployment or early retirement, it may hinder the possibilities for cancer survivors to continue working with employers reluctant to hire older, usually more expensive workers (Ahn et al., 2009).

In contrast, higher family income was a facilitating factor in RTW at six-months and one-year post-treatment. Echoing previous evidence, higher family income often implies higher educational attainment associated with types of work that are less physically demanding (Drolet et al., 2005; Ahn et al., 2009), which in turns facilitated RTW. Moreover, high earners may experience greater loss of income than low earners, and government support for retirees, in Hong Kong at least, provides little security. Blue collar work and service-oriented jobs often entail more manual work and labor that are physically demanding. (Wolvers et al., 2018).

Marital status and gender were unrelated to RTW status. In most Asian cultures, women are traditionally expected to work in caring for their family while men are usually the major breadwinner of the family (Lee et al., 2008). Marriage may therefore provide financial support and reduce the urge or need to return to work among married female patients in Asia (Lee et al., 2008). However, this might be less significant in Hong Kong where a substantial proportion of women (45%) is engaged in the labor force (Secretariat Legislative Council, 2019). As in Western settings (Drolet et al., 2005), neither marital status nor gender significantly differentiated returned to work from unemployed in the univariate analysis; thus, these factors were excluded from the multivariate analyses.

We also examined if clinical factors predicted RTW status. Compared with breast cancer survivors, head and neck cancer survivors were more likely to be still on sick leave early in the recovery period. This concurs with the findings reported in previous studies that RTW rates differ by cancer type and treatment type (Cooper et al., 2013; Muijen et al., 2013; Arfi et al., 2018). For example, the side effects of nasopharyngeal cancer affect swallowing and speech, and these as well as treatment-induced hearing loss can hinder performance in most types of work (Lee et al., 2007). These impairments may erode a patients’ confidence and ability needed for returning to work, being compounded by communication difficulties and impaired social functioning among these patients (Vartanian et al., 2006), and raise the potential for shame and embarrassment as a result. Previous studies documented that cancer survivors who had completed chemotherapy were more likely to have had longer sick leave (Balak et al., 2008; Fantoni et al., 2010; Gordon et al., 2014) and a greater probability of being unemployed (Vartanian et al., 2006; Johnsson et al., 2011; Gordon et al., 2014). Cognitive impairments affecting verbal and executive functioning, information processing speed, memory deficiency, and decision making, all of which are key elements requirements involved in working have been attributed to chemotherapy (Munir et al., 2010; Myers, 2012; Kamal et al., 2017). Our findings identified having chemotherapy as a factor that was significantly associated with delayed return to work at six-months post-treatment. Of course, this may also reflect differences in disease or treatment aggressiveness.

The current study revealed that poor physical functioning may delay work resumption among cancer patients. At six-months post-treatment, those on sick leave or unemployed perceived themselves to have poorer physical functioning than did those who had returned to work. Previous studies have reported that degree of physical and psychological symptoms such as fatigue, loss of appetite, depressive symptoms, and higher anxiety levels predict employment status (Balak et al., 2008; Lee et al., 2008; Johnsson et al., 2011; Muijen et al., 2013). Of interest, in our study, those on sick leave or who were unemployed did not report significantly more physical symptom or psychological distress than those who had returned to work. However, these results must be treated with caution as low statistical power arising from the small number (*N* = 13) of those on sick leave at one-year post-treatment may account for the insignificant associations. Alternatively, these discrepancies may suggest that at least some of the concerns of returning to work in the Hong Kong Chinese population may differ from those observed in western populations. For instance, employers may discourage RTW in older cancer survivors when a younger, healthier (and cheaper) replacement is available. Further studies that explore other work-related factors such as work conditions, job satisfaction, and the limited sickness benefit or absence of pension provision different populations are needed (Amir and Brocky, 2009; Mehnert, 2011).

We observed an association between illness perception and RTW status at six-months post-treatment by revealing those who were unemployed after cancer diagnosis were more likely to perceive greater illness impacts than those who returned to work. This finding may potentially reflect that those who were unemployed perceived themselves as experiencing more severe illness impacts than those on sick leave or who had returned to work. Therefore, instead of taking sick leave, they may have decided to resign or retire early in the illness trajectory. Those on sick leave may have considered the illness impacts they were experiencing as temporary and remediable, given time. This highlights the importance of early screening in occupational rehabilitation settings for high-perceived illness impacts, in order to identify patients prevented from returning to employment thereby. This can help to promote return to work as early as possible after cancer diagnosis. Occupational rehabilitation can help patients to recover or optimize their functioning in a shorter period of time by helping involuntarily unemployed patients to resolve symptoms and build confidence and therefore be more likely to re-enter the workforce at an early stage of cancer, thereby further facilitating recovery. Those patients who had resumed work may also experience specific needs and concerns in relation to work resumption. As observed in the current study, patients in the RTW groups, especially those who reported less absenteeism had perceived more unmet needs in health system and information than those who had not returned to work at six-months post-treatment, suggesting that the provision of healthcare service information to those who have planned to resume work after cancer diagnosis by healthcare professionals may also play a facilitating role in promoting and sustaining RTW in this population.

In terms of work productivity and activity impairment at one-year post-treatment, participants diagnosed with gynecological cancer were found to be more prone to suffer from absenteeism when compared to breast cancer patients. In a study of clinical predictors of RTW by cancer type, gynecological cancer patients receiving radiotherapy reported more stiffness in joints and muscles, localized swelling and skin soreness in the affected area than did breast cancer patients (Cooper et al., 2013). Such patients with impaired physical wellbeing may be more likely to report in reduced work hours (Mamguem Kamga et al., 2020).

Absenteeism was also positively associated with greater unmet sexuality needs in the current study. Patients with greater unmet sexuality needs at six-months post-treatment were likely to report longer time off work at one-year post-treatment. This finding was further supported by a recent systematic review of 37 studies among gynecological cancer patients, which suggested that work restrictions due to health condition was a risk factor of unmet supportive care needs including sexuality unmet needs (Beesley et al., 2018). However, the results must be interpreted with caution since the overall unmet needs on sexuality were relatively low in the current study, with a mean score of 6.32. Alternatively, a possible explanation for the positive association between absenteeism and unmet sexuality needs may be that greater unmet sexuality needs reflect lower perceived social/family support (McDowell et al., 2010), in which has been proposed as crucial individuals’ personal resources for coping with and handling the process of RTW (Englund et al., 2016). Without a supportive environment, cancer patients are likely to experience work exhaustion (Shin et al., 2021), potentially leading to increased absenteeism. Future studies need to further explore this link.

Presenteeism and reduced work productivity were positively associated with depressive symptoms, physical symptom burden, and negative illness perceptions. Studies have revealed that after 4–10 years from cessation of primary cancer treatment, one-third of breast cancer patients continued to report fatigue, with over 20% experiencing depression (Bowen et al., 2007). Physical and psychological symptom distress tends to cluster, affecting work productivity for years after primary treatment (Hansen et al., 2008). A recent study of Chinese women with breast cancer reported higher levels of anxiety, more depressive symptoms, and work productivity loss than in healthy individuals (Zeng et al., 2015), which echoed previous reports on Western population (Calvio et al., 2010). A local Hong Kong Chinese population study examining the impact of psychological symptom distress on work productivity also reported a positive association between anxiety levels and work productivity loss in breast cancer survivors (Cheng et al., 2016). Our findings are consistent with previous evidence that unmanaged physical symptom distress and psychological distress hindered work productivity, with the exception of an insignificant association found between anxiety level and work productivity loss.

This study also sheds light on the role of illness perceptions in work productivity. To our knowledge, this is the first study to describe such association in cancer populations. Patients who reported more negative illness perceptions also experienced more work productivity loss possibly because they perceived greater illness impacts or more negative symptom experiences and greater illness concerns when they did return to work. Those who returned to work were more likely to realize any impairment in their work productivity, something that may be less apparent to those on sick leave or unemployed.

Activity impairment was greater in those working in service-oriented jobs than in office workers. Service-oriented jobs may require long working hours in different work locations, and a higher likelihood of commission-based instead of fixed-salary remuneration than office workers. Hence, service-oriented workers, like manual workers, face greater job demands that can reveal reduced capacity for sustained work. Additionally, for those who did not return to work after cancer diagnosis, activity impairment was likely evidenced in daily activities. In a previous study that examined the impacts of cancer and its treatments in terms of daily activities, 65% of patients considered at least some daily and self-care activities (ADLs) that may be perceived as less physically demanding, such as walking around at home, taking a bath, getting dressed, and managing light house work as “troublesome” (Peuckmann et al., 2009). Non-Caucasian cancer survivors also report significantly more fatigue when performing housework compared to population-based controls (Lee et al., 2008). Therefore, rebuilding activity capacity through rehabilitation is a vital step in promoting the renormalization of cancer patients for both occupational and ADL reasons.

### Study Limitations

Several limitations of the present study should be noted. All patients were recruited from government-funded public hospitals; therefore, findings might not be generalizable to private settings, where patients are more likely to have a higher socioeconomic status and different health-seeking behaviors, thus, differences in unmet needs and occupational demands. However, most cancer care is delivered through public hospitals in Hong Kong. Therefore, this study is able to present a representative picture of the RTW phenomenon among Hong Kong cancer patients. Also, the effect of adjuvant therapy on RTW during cancer treatment may be implicated because, while patients had finished primary treatments at the time of recruitment some patients continue to receive therapies (e.g., hormonal therapy) for up to 5 years post-diagnosis, which may impede recovery. This, perhaps, has contributed to delayed RTW at baseline assessment. Furthermore, it is important to note that this study did not compare the rate of unemployment among cancer survivors and healthy controls, which may contribute to a more accurate description of the impact of cancer and its treatments among cancer survivors. Lastly, this study did not account for the role of cognitive impairment on RTW, work productivity, and activity impairment. Previous studies identified that cognitive impairment, associated with chemotherapy, may hinder work ability, and thus delay work resumption (Munir et al., 2010). This study was part of a longitudinal project on cancer survivorship, particularly in relation to supportive care needs, so cognitive impairment was not included as a potential correlate.

## Conclusion

The impact of cancer and its treatments hinder RTW and work productivity. Cancer survivors face various challenges in RTW and work productivity, especially at the initial recovery stage as indicated by a relatively lower RTW rate at baseline. However, locally, cancer rehabilitation is lacking and not well integrated into oncology clinical practice. Developing an evidence-based rehabilitation intervention to support cancer survivors in returning to work should be considered a research priority.

This study enables us to identify specific RTW and work productivity issues faced by cancer patients with different RTW status. Cancer survivors who had more physically demanding jobs and poorer physical functioning delayed work resumption, while unmanaged physical symptom and psychological distress hindered work productivity. The findings allow healthcare service providers to develop a risk profile for identifying patients who are at higher risk in delayed work resumption and work productivity loss, so as to offer timely referral for occupation rehabilitation at earlier stages of cancer rehabilitation. For example, clinicians could identify the subset of affected patients with early screening of physical functioning. With early interventions that aim to recover and achieve desirable functioning to facilitate early RTW, given adequate labor market opportunities, those who were unemployed and on sick leave involuntarily are more likely to remain or re-enter the workforce after their cancer diagnosis.

## Data Availability Statement

The raw data supporting the conclusions of this article will be made available by the authors, without undue reservation.

## Ethics Statement

The studies involving human participants were reviewed and approved by the Institutional Review Board of the University of Hong Kong/Hospital Authority Hong Kong West Cluster (Ref: UW10-203). The patients/participants provided their written informed consent to participate in this study.

## Author Contributions

SS and DN contributed equally and drafted the manuscript. SS, DN, QL, and WL performed the statistical data analysis. IS, KC, CL, AN, WS, WC, and VL coordinated the recruitment sites. WL and RF contributed to manuscript revision and approved the final submitted version. All authors have read and agreed to the published version of the manuscript.

## Funding

This study was funded by Hong Kong Cancer Fund.

## Conflict of Interest

The authors declare that the research was conducted in the absence of any commercial or financial relationships that could be construed as a potential conflict of interest.

## Publisher’s Note

All claims expressed in this article are solely those of the authors and do not necessarily represent those of their affiliated organizations, or those of the publisher, the editors and the reviewers. Any product that may be evaluated in this article, or claim that may be made by its manufacturer, is not guaranteed or endorsed by the publisher.
